# The Effect of Mechanical Stress on Plant Susceptibility to Pests: A Mini Opinion Review

**DOI:** 10.3390/plants9050632

**Published:** 2020-05-14

**Authors:** Catherine Coutand

**Affiliations:** INRAE ur 1115 Plantes et Systèmes de culture Horticoles (PSH), CEDEX 9, 84914 Avignon, France; catherine.coutand@inrae.fr

**Keywords:** Mechanical stress, plant susceptibility, plant immunity, defense gene, hormone, pest, pathogen

## Abstract

Plants are subject to multiple pest attacks during their growing cycle. In order to address consumers’ desire to buy healthy vegetables and fruits, i.e., without chemical residues, and to develop environment-friendly agriculture, major research efforts are being made to find alternative methods to reduce or suppress the use of chemicals. Many methods are currently being tested. Among these methods, some are being tested in order to modify plant physiology to render it less susceptible to pathogen and pest attacks by developing plant immunity. An emerging potentially interesting method that is being studied at this time is mechanical stimuli (MS). Although the number of articles on the effect of MS on plant immunity is still not large, it has been reported that several types of mechanical stimuli induce a reduction of plant susceptibility to pests for different plant species in the case of wounding and non-wounding stimuli. This mini review aims to summarize the knowledge available at this time by raising questions that should be addressed before considering MS as an operable alternative method to increase plant immunity for crop protection.

## 1. Introduction

Crops are subject to multiple pest attacks during their vegetative and fruiting periods. To respond to consumer demand for chemical-free fruits and vegetables, authorities are changing policies in order to reduce chemical use for crop protection. Major research efforts are being developed to find alternative methods to reduce or suppress the use of chemicals and to design innovative crop systems. One avenue currently being explored is to tune the physiological state of plants to increase their natural immunity. Many methods are being tested at this time, including those involving irrigation tuning (e.g., [1,2]), N supply tuning (e.g., [2,3]), companion plants (see [4] for a review), plant resistance inducers (PRI) ([5]), and so on, to increase plant resistance through increased natural immunity [6]. An emerging potentially interesting method that is currently being studied is mechanical stimuli (MS). This mini review aims to summarize the knowledge available at this time concerning the effects of MS on plant susceptibility to pests. Since the term “pests” encompasses herbivores, sucking feeding insects, fungi, and weeds, an effort has been made in this review to distinguish between the different types of pests whenever possible. The term MS can cover widely different types of stimuli, which are poorly quantified at this time. From our point of view, studies on MS considering it as an innovative method to increase plant immunity would be greatly improved if the question of MS quantification was addressed. A part of this review thus explains the benefits of MS quantification and the way it could be better understood. Another important and interesting issue is to decipher the genetic and metabolic pathway that is involved in MS perception in plants and how it can modify plant physiology to create less susceptible plant phenotypes in the event of pest attacks. The second part is thus dedicated to the different components of plant physiology that are involved or could be involved in MS-induced immunity. This review concludes with other important aspects that should be considered before considering MS as an operable method for the design of innovative cropping systems.

## 2. Mechanical Stimuli Have an Impact on a Wide Variety of Plants, Pests and Pathogens

The term “mechanical stimuli” encompasses a wide range of types. For example, some are wounding and others not. Among wounding MS, some can be artificially applied to plants and some result from a herbivore or necrophytic fungi attack. In this review, only artificial wounding was considered. Despite different types of MS, it appears that MS generally leads to a reduction in plant susceptibility to pests. It should be noted that almost all of the studies on the effects of MS on plant susceptibility concern herbivores and necrophytic fungi.

In the case of wounding stimuli, local wounding in Arabidopsis strongly inhibited the hyphal growth of *Botrytis cinerea* [7] but only temporarily. In the case of non-wounding mechanical stimuli applied to plants, the literature is more abundant. Pioneering studies have demonstrated that wind-induced mechanical stimuli (produced by fans) applied on bean plants reduced the egg production and growth of spider mites on foliar disks of mechanically-stimulated plants, as well as on whole plants [8]. Touch was shown to reduce the weight of *Trichoplusia ni* larvae in Arabidopsis [9]. Interestingly, in addition to the efficient effect of MS on herbivores, it was recently reported that touch also decreased the attractivity of aphids in maize and bean plants [10]

MS also provide increased immunity in the case of necrophytic fungus: touch was shown to reduce the lesion size on Arabidopsis plants due to *Botrytis cinerea* [10]. Gentle leaf rubbing between the fingers induced a reduction of symptoms due to *Botrytis cinerea* in Arabidopsis [11]. The positive effect of gentle rubbing was also demonstrated on strawberry plants, where it reduced lesion size due to *Botrytis cinerea* [12]. 

These results obtained in different plant species and with different pests seem to indicate that mechanical stimuli may act as an efficient method to increase plant immunity over a broad spectrum of “wounding” pests. It would be very interesting to determine whether or not the spectrum of action of MS is limited to “wounding” pests and sucking feeding insects or if MS could also be of interest in the case of biotrophytic fungi. Nevertheless, before mechanical stimuli can be used as a method in the design of innovative crop systems, several questions must be answered.

## 3. Quantifying Mechanical Stimulation in the Case of Non-wounding Stimuli: Spatial and Temporal Aspects

The application of MS raises questions about the intensity of MS, the time of its application and the frequency at which it should be used to obtain a long-lasting effect. It thus appears that the application of MS covers spatial and temporal aspects. The spatial aspect includes the effect of the mechanical stimulus on the plant’s mechanical state, the stimulation of all or part of the plant, and the possible or unsystemic effect of MS. The temporal aspect covers the relative kinetics of MS application vs. the kinetics of infection, the duration of the effect of MS on plant immunity, which in turn determines the frequency at which MS should be applied to obtain long-lasting effects, i.e., that cover the period when the crop is subjected to the targeted pest.

### 3.1. Quantifying the Mechanical State of Plants Due to Mechanical Stimulus

Existing studies are generally semi-quantitative because the mechanical stimulus is quantified as the number of touches or rubs, for example [9,11,12]. It was shown on Arabidopsis that there is an effect of the number of rubs on the level of symptoms: up to five rubs were not sufficient to induce a significant decrease in symptoms, but five to ten rubs led to a gradually significant decrease of the symptoms due to *Botrytis cinerea,* and ten rubs led to a reduction of lesion size of more than 90% in comparison to unstimulated plants [11]. Despite the fact that this study indicated a quantitative effect of mechanical stimuli, the stimulus is probably partly dependent on the experimenter, and thus not easily reproducible. Rubs might also be a bit difficult to apply in cropping systems. The lack of quantification of MS also makes it difficult to compare different studies, e.g., to assess whether or not some genetic inter- or intra-plant species variability exists, as in the case of thigmomorphogenesis using MS quantification [13]. Finally, the lack of quantification of MS does not allow us to compare the efficiency of MS on the different types of pests, an important aspect of crop protection management over the entire growth cycle when the crop undergoes successive attacks, and because protection from one pest may favor the development of another. However, studies on MS-induced plant resistance indicate a positive effect of “light” MS. 

To our knowledge, no study has yet been carried out that actually controls mechanical stimuli and that quantifies changes in the mechanical state of the plant induced by the stimuli related to the effect of mechanical stimuli on plant susceptibility to pests. Interestingly, studies of thigmomorphogenesis performed in the 2000s were dedicated to quantifying the change in the mechanical state of the plant induced by the stimulus and to linking it to induced growth changes [14,15]. These studies used bending as a mechanical stimulus because of its similarity to stimuli induced by the wind sway of stems, and because its nature makes it possible to quantify it in a simple way using structural mechanics equations from beam theory (see [15]). The mechanical state of a bent stem can be defined by static variables such as force (global scale) or bending moment and longitudinal stress (local scale), and by kinetic variables such as lateral displacement at the tip of the stem (global scale), curvature, and longitudinal strain (local scale) [15]. These studies demonstrated that plants do not perceive force or stress but, instead, strain. The S3m model of mechanoperception was derived from these studies; each little piece of bent tissue triggers a little signal s proportional to the strain it is subjected to, and when these signals are added up, they form the thigmomorphogenetic signal at the stem scale [15]. The S3m model of mechanoperception is defined by Equation (1) [15]:

(1) $$S_{i,l\left(\varepsilon \right)}={∭}_{Vs}k_{0\left(\zeta ,y,z\right)}\times \left(\varepsilon_{\left(\zeta ,y,z\right)}-\varepsilon_{0}\right)\times dV$$

With *S_i_* the global internal morphogenetical signal, *ε* the longitudinal strain, ε_0_ the threshold strain, *Vs* the cell volume, *k_0_* the mechanosensitivity, ζ the distance from the apex, and (*y*,*z*) the position of the tissue elements across the stem cross section.

This finding made it possible to control applied bending in an easy way by bending plant stems along guides of known curvature [13,16] so as to control the applied sum of strain. The framework of the S3m mechanoperception model could be an interesting tool to perform a quantitative analysis of the effect of mechanical stimuli on plant susceptibility to pests in order to establish the quantitative link between the applied stimulus and the level of symptoms, and probably also to decipher the involved metabolic and/or gene pathway. Indeed, if there is a quantitative link between MS and the phenotype, this quantitative link must be maintained throughout the whole metabolic and genetic pathway; otherwise, the quantitative information would be lost.

### 3.2. Timing between the Application of Mechanical Stimulation and Inoculation

The time between the application of mechanical stimuli and the time of inoculation indicates the effect of mechanical stimuli alone on the plant physiological status and its effect on the infection phase. This aspect is actually poorly covered in the literature. To our knowledge, only the study conducted by Chassot et al. [7] has focused on the lag time between MS and inoculation, and the authors reported that wounding had an efficient effect on infection by *Botrytis cinerea* for about 48 h. It should be noted that in studies where this data is available, the time between MS and the time of inoculation is variable; in the case of *Botrytis cinerea*, it is 10 min in [11] and 30 min in [12]. In the case of insects, the lag time was 24 h [10]. 

### 3.3. Persistence of the Effect of Mechanical Stress on Plant Susceptibility to Pests

The time of persistence of the effect can be defined as the time that the effect of mechanical stimuli lasts once the mechanically-stimulated plant has been inoculated. It thus measures the lasting effect of mechanical stimuli on the development of symptoms and quantifies the lasting effect of the MS stimulus*inoculation interaction. It can only be estimated by comparing the development of symptoms between mechanically-stimulated and control plants as a function of time. The study conducted by Chehab et al. [9] reported that the reduction of lesion size due to *Botrytis cinerea* was detectable two days post-inoculation (dpi) and was still visible at three dpi. In strawberry plants, the differences in lesion size between MS-treated and control plants was not significant at 2 dpi, but became significant at 4 dpi and even greater at 6 dpi [12]. This result is interesting because it paves the way for a frequency choice of MS application on plants. Indeed, considering MS as one of the possible options for increasing plant immunity will probably require successive applications of MS to maintain the MS effect over a sufficient length of time. This aspect raises two questions. Can the effect of MS on immunity be maintained by successive applications of MS? If the answer is yes, the second question is: what is the adequate frequency of stimulation? In the field of thigmomorphogenesis, it has been shown that repeated MS at a low frequency of one bending per day leads to a desensitization of plants to bending as of the third day [17]. It was also shown that a rest period of about ten days is required by the plant to recover its full sensitivity to MS [17]. The results obtained in [12] and [17] suggest that to maintain MS-induced plant immunity, a frequency of MS application every 7–10 days should be sufficient. In addition to the quantitative aspects, it is crucial to better understand the metabolic and genetic changes induced by MS and how they positively affect plant immunity.

## 4. Pathways Involved in Mechanically-Induced Resistance to Pests

When plants are exposed to herbivores or a fungus, they deploy defense reactions. For example, in the case of an attack by a necrophytic fungus (e.g., *Botrytis cinerea* or *Sclerotinia sclerotiorum*), plants deeply modify their physiology [18] by: (i) modifying their redox cell status via NO2- or reactive oxygen species (ROS) [19,20,21,22]; (ii) locally or systemically synthesizing antifungal compounds via a complex hormonal pathway relying on jasmonic acid (JA), salicylic acid (SA), abscisic acid (ABA), and ethylene (ET) (see [23,24]); and (iii) reinforcing cell walls [19,24]. Concerning changes in plant hormonal status after a pest attack, the general mechanism is the activation of the SA pathway in the case of biotrophic fungus (but not exclusively), and the involvement of the ET/JA pathway in the case of a necrophytic fungus or herbivore attack [25]. 

### 4.1. Oxidative Stress

Some studies have demonstrated that wounding MS induce the production of ROS [26,27]. The study conducted by Benikhlef et al. [11] revealed that non-wounding stimuli led to the formation of ROS as well and that the ROS signal appeared to be quantitatively linked to the number of rubs. The production of ROS was shown to be a *sine qua non* condition for the decrease of tomato susceptibility to *B. cinerea*. Since oxidative burst is rapidly encountered in plants after MS and because studies that demonstrated the efficiency of MS to reduce disease symptoms were performed with an inoculation time of no longer than 30 min [11], this suggests that the oxidative burst in MS plants does not prevent infection but slows down the development of symptoms. This result is very interesting and intriguing as well. Indeed, it is thought that necrophytic fungi development *in planta* benefits from the oxidative burst it generates in the plant after attack [28,29]. Consequently, one could expect that MS-induced oxidative burst could be of benefit to the fungus as well. Interestingly, the study conducted by Tomas-Grau [12] demonstrates that in strawberry, the oxidative burst is extended to the whole leaflet in control plants, whereas it is restricted to the lesion area in the case of MS-treated plants. In addition, this study also revealed an up-regulation of the FaCAT gene that encodes a catalase in the strawberry plant [30], which could explain the low H2O2 accumulation observed 48 h after MS treatment.

### 4.2. Hormones and Defense-Related Genes

MS applied to plants are known to induce changes in plant hormonal status (see [31] for a review). However, further research is still required. To summarize:

Touch has been shown to increase the level of lipoxygenase (LOX) transcripts, as well as other genes implicated in the JA biosynthetic pathway [32,33,34]. A possible scenario could be an increase in cytosolic Ca2+ levels, which, in turn, might be responsible for the activation of phospholipase D (PLD) that releases free polyunsaturated fatty acids from membranes that then activate lipoxygenase (LOX) to trigger the JA biosynthetic pathway. MS have been shown to induce ethylene (ET) levels in MS-treated plants in different species [35], and it has been shown that MS mimics changes due to ET exposure. However, the ET pathway does not appear to be essential for plant growth responses to MS and is not the earliest metabolic actor within the mechanotransduction pathway [32,36]. Concerning abscisic acid (ABA), it has been shown that MS-treated plants accumulate ABA [37,38], but further studies are required to address the role of ABA in the mechanotransduction pathway [32]. Brassinosteroids (BRs) are thought to be involved in thigmomorphogenesis. Indeed, it has been shown that plant exposure to BRs results in an increase in transcripts of TCH4, which is one of the TOUCH genes [32].

The study conducted by Cipollini and Redman [39] revealed that that MS effect could be mimicked by application of JA. Wind-induced MS led to an activation of peroxidase (POD) and cinnamyl alcohol deshydrogenase (CAD) activities in tomato [40], two enzymes of the phenylpropanoid pathway generally associated with JA signaling. Nevertheless, the study did not investigate the enzyme activities in MS+infection treated plants. The study conducted by Chehab on Arabidopsis [9] revealed that the JA pathway is implicated within the perception-transduction pathway of the action of MS on plant susceptibility to *Botrytis cinerea*. In contrast, the study conducted by Benikhlef et al. [11] demonstrated with transformed plants that the JA pathway is not required for the positive effects of MS to increase plant immunity. This apparent discrepancy might be explained by two hypotheses: (i) in these two studies, the Arabidopsis lines were not the same (except for the Col 0 line used for controls), so that the pathway involved might depend on the plant genotype; and (ii) the second hypothesis, which is not exclusive of the first one, might be that in order to have effective immunity, plants might have different efficient pathways and that one will be favored if another is impaired. 

Concerning ethylene (ET), it is known to be an important actor in plant response against pathogens [41]. The study conducted by Chenab et al. [9] on *Arabidopsis thaliana*, revealed that the ET pathway is not required for the perception of mechanical stimuli. However, the ACS gene, coding for a key enzyme of ET biosynthesis, was found to be upregulated in MS-treated plants in *Vigna radiata* [42]. The study conducted by Tomas-Grau et al. on strawberry plants [12] revealed that the FvACS1 gene was highly upregulated in MS-treated plants 48 h post-MS treatment in comparison to control plants. It might therefore be possible that ET is involved in a plant defense pathway induced by MS, even if it is not implicated in the MS perception pathway. The combination of biotic and abiotic stress revealed complex interactions within the hormonal pathways [43,44,45]. Nevertheless, up to now, there is no study on the effect of MS on plant immunity that reveals the involvement of SA, perhaps because the current studies were performed with necrophytic pathogens only. Nevertheless, a lead that might be useful to follow up is the study of expression of WRKY genes, generally associated with the SA pathway. Indeed, WRKY genes appear to play a role at cross talks between biotic and abiotic stresses. Consequently, “as the regulation of plant responses to multiple stress relies on tightly regulated and highly dynamic regulatory networks and that combined stresses do not have compulsorily additive effects, WRKY as well as defense genes should be studied under individual stresses as well as in combination” [46]. In the studies currently available in the literature on the effect of MS on plant immunity, the expression of defense genes appears to only have been studied in the case of MS-non-inoculated treated plants but not on MS-inoculated treated plants. Indeed, it is interesting to note that the study conducted by Molloreau et al. [5] on the efficiency of PRI compounds to fight *Erwinia amylovora* on apple compared the expression level of defense genes between control plants (NO PRI, no H2O2; H2O2 treatment is used to mimic a fungus attack), PRI-no H2O2 treated plants, PRI+H2O2 treatment and no PRI+H2O2 treatment, and revealed a priming effect of PRI; the PRI alone does not induce defense genes but stimulates the expression of defense genes in the case of H2O2 treatment. In the case of MS, it might be possible that at least some defense genes involved in the pathway are triggered via a priming effect so that they cannot all be revealed by the study of defense gene expression in MS-uninoculated treated plants alone.

Finally, PR genes are involved in the plant defense pathway and are upregulated after pathogen attack [47,48,49]. It is interesting to note that the study conducted by Lee et al. [33] showed that 14 PR genes were upregulated in MS-treated plants compared to the controls. In acacia, it was shown that R protein genes were upregulated in young trees subjected to bending within 30 min after stress treatment [50]. In strawberry, it was shown that several PR genes are upregulated after MS treatment *in planta* and associated with plant immunity towards *B. cinerea*. PR1 was highly upregulated 48 h post-MS treatment [12], extending the result obtained with MS in parsley cell culture [51]. In strawberry plants, other PR2 and PR3 genes were also shown to be upregulated after MS treatment [12]. PR2 and PR3 genes encode for β-1,3-endoglucanases and chitinase type II (Chi2) enzymes involved in cell-wall modifications [52]. Nevertheless, it should be noted that the overexpression of PR genes is unlikely to explain the wounding-induced resistance in tomato [53].

From a kinetics point of view, the study conducted by Tomas-Grau et al. [12] on strawberry plants demonstrated that TOUCH genes are upregulated as early as 30 min after rubbing was applied, whereas defense genes were upregulated only 48 h after MS were applied. Interestingly, the defense genes were upregulated before significant differences in symptoms could be observed between mechanically-treated and control plants. To our knowledge, concerning *B. cinerea* and in the case of non-wounding stimuli, there is no study that investigated the effect of inoculation at times longer than 30 min after MS application. It would be interesting to inoculate plants once defense genes are upregulated by MS in order to see if the MS-induced defense genes can counteract the development of the symptoms induced by the fungus, but when inoculation is performed after the MS-induced oxidative burst. 

In addition to these MS effects on plant immunity in the case of necrophytic pathogens and herbivores, this mini review concludes with recent knowledge obtained about the effect of MS on sucking feeding insects via organic volatile compound (VOC) emission. 

### 4.3. Volatile Organic Compounds (VOC)

The emission of volatile organic compounds (VOC) has been shown to play a role in plant-insect interactions (see [4] for a review). Some VOC emitted by companion plants are effective for reducing aphid attractivity in pepper [54,55]. However, VOC emitted by the crop itself if mechanically stimulated can also be effective. The study conducted by Markovic et al. [10] investigated the possible role of MS on plant volatile compounds and their effects on aphid attractivity in maize and bean. The results revealed that MS modified the volatile profile of touched plants and that these changes were sufficient to decrease the attraction of aphids to touched plants in comparison to control plants. The nature of volatiles between touched and untouched plants was studied and revealed that the volatile profiles were discriminated by (E)-nerediol and (E)-beta-caryophyllene in maize, and 6-methiyl-5hepten-2-one and an unidentified sesquiterpene in bean plants [10]. These studies suggest that MS could be used to stimulate VOC emission by mechanically-stimulating the crop itself and/or could be used to stimulate the VOC emissions of MS-stimulated companion plants.

## 5. Conclusions

**Figure 1:**
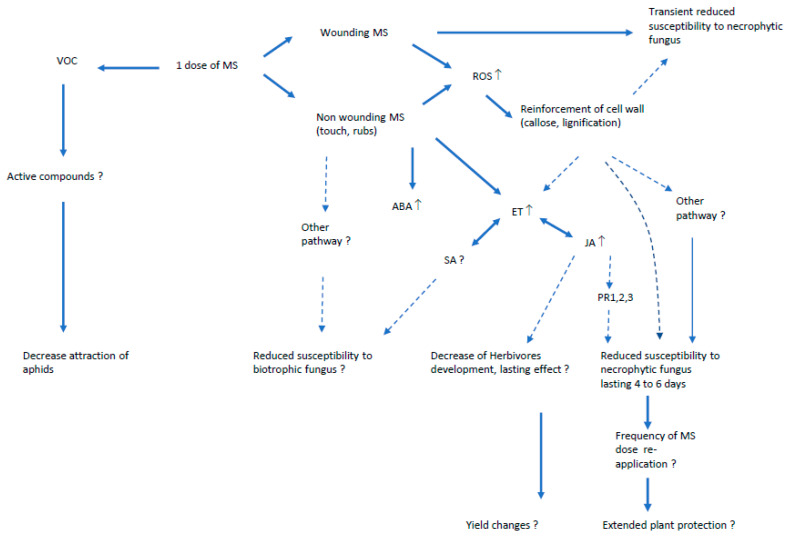
Summary scheme of the effect of mechanical stress (MS) in the objective of crop protection. VOC = volatile organic compounds; ROS = reactive oxygen species; ABA = abscissic acid; ET = ethlyne pathway; JA = jasmonate pathway; SA = saicylic acid pathway; PR = defense related proteins.

Knowledge about the effects of MS on the plant immunity pathways is still partial and at its beginning (Figure 1), and thus needs to be further developed. The inclusion of MS as an alternative method in the design of innovative crop systems has not yet been reached, but very probably it is likely that the quantification of MS, combined with biotests over longer periods to assess the effective frequency of MS application, will help to determine the best conditions for its use as an interesting method for crop protection. Moreover, the combination of MS quantification with the use of omic tools will help us to acquire the required knowledge for a better understanding of the pathway(s) involved.

Another important aspect that must be considered is the positive or negative impact of MS on fruit quality and yield. To our knowledge, the impact of MS on crop yield has never been assessed and will probably depend on the frequency at which it should be applied. Concerning the effects of MS on fruit quality, a study revealed that MS favors the production of antioxidant compounds that are known to be of interest for human health [56]. Nevertheless, more results are necessary to confirm these first preliminary results.

Generally speaking, a single method only provides a partial resistance effect, so that when we have acquired sufficient knowledge about MS and confirmed the potentiality of MS as a method to stimulate plant immunity, the second step will be to study MS in combination with other methods to design innovative crop systems.

## References

[1] Achuo E.A., Prinsen E., Hofte M. (2006). Influence of drought, salt stress and abscisic acid on the resistance of tomato to botrytis cinerea and oidium neolycopersici. Plant Pathol..

[2] Lecompte F., Nicot P.C., Ripoll J., Abro M.A., Raimbault A.K., Lopez-Lauri F., Bertin N. (2017). Reduced susceptibility of tomato stem to the necrotrophic fungus Botrytis cinerea is associated with a specific adjustment of fructose content in the host sugar pool. Ann. Bot..

[3] Lecompte F., Abro M.A., Nicot P.C. (2013). Can sugar mediate the effect of nitrogen fertilization on lettuce susceptibility to two necrotrophic pathogens: Botrytis cinerea and Sclerotinium sclerotinium?. Plant Soil.

[4] Ben-Issa R., Gomez L., Gautier H. (2017). Companion Plants for Aphid Pest Management. Insects.

[5] Marolleau B., Gaucher M., Heintz C., Degrave A., Warneys R., Orain G., Lemarquand A., Brisset M.-N. (2017). When a Plant Resistance Inducer leaves the lab for the field: Integrating ASM into routine apple protection practices. Front. Plant.

[6] Walters D.R., Ratsep J., Havis N.D. (2013). Controlling crop diseases using induced resistance: Challenges for the future. J. Exp. Bot..

[7] Chassot C., Buchala A., Schoonbeek H.-J., Metraux J.-P., Lamotte O. (2008). Wounding of Arabidopsis leaves causes a powerful but transient protection against Botrytis infection. Plant J..

[8] Cipollini D.F. (1997). Wind-induced mechanical stimulation increases pest resistance in common bean. Oecologia.

[9] Chehab E.W., Yao C., Henderson Z., Kim S., Braam J. (2012). Arabidopsis Touch-Induced Morphogenesis Is Jasmonate Mediated and Protects against Pests. Curr. Biol..

[10] Markovic D., Glinwood R., Olsson U., Ninkovic V. (2014). Plant response to touch affects the behaviour of aphids and ladybirds. Arthropod-Plant Interact..

[11] Benikhlef L., Floriane L’Haridon F., Abou-Mansour E., Serrano M., Binda M., Costa A., Silke Lehmann S., Métraux J.-P. (2013). Perception of soft mechanical stress in Arabidopsis leaves activates disease resistance. BMC Plant Biol..

[12] Tomas-Grau R.H., Requena-Serra F.J., Hael-Conrad V., Martinez-Zamora M.G., Guerrero-Molina M.F., Diaz-Ricci J.C. (2017). Soft mechanical stimulation induces a defense response against *Botrytis cinerea* in *s*trawberry. Plant Cell Rep..

[13] Coutand C., Chevolot M., Lacointe A., Rowe N., Scotti I. (2010). Mechanosensing of stem bending and its interspecific variability in five neotropical rainforest species. Ann. Bot..

[14] Coutand C., Moulia B. (2000). Biomechanical study of the effect of a controlled bending on tomato stem elongation: Local strain sensing and spatial integration of the signal. J. Exp. Bot..

[15] Moulia B., Coutand C., Julien J.-L. (2015). Mechanosensitive control of plant growth: Bearing the load, sensing, transducing and responding. Front. Plant Sci..

[16] Coutand C., Martin L., Leblanc-Fournier N., Decourteix M., Julien J.-L., Moulia B. (2009). Strain Mechanosensing Quantitatively Controls Diameter Growth and PtaZFP2 Gene Expression in Poplar. Plant Physiol..

[17] Martin L., Leblanc-Fournier N., Julien J.-L., Moulia B., Coutand C. (2010). Acclimation kinetics of physiological and molecular responses of plants to multiple mechanical loadings. J. Exp. Bot..

[18] Windram O., Madhou P., McHattie S., Hill C., Hickman R., Cooke E., Jenkins D.J., Penfold C.A., Baxter L., Breeze E. (2012). Arabidopsis Defense against Botrytis cinerea: Chronology and Regulation Deciphered by High-Resolution Temporal Transcriptomic Analysis. Plant Cell.

[19] Asselbergh B., Curvers K., Franca S.C., Audenaert K., Vuylsteke M., Van Breusegem F., Hoefte M. (2007). Resistance to botrytis cinerea in sitiens; an abscisic acid-deficient tomato mutant; involves timely production of hydrogen peroxide and cell wall modifications in the epidermis. Plant Physiol..

[20] Mengiste T. (2012). Plant immunity to necrotrophs. Annu. Rev. Phytopathol..

[21] Perchepied L., Balague C., Riou C., Claudel-Renard C., Riviere N., Grezes-Besset B., Roby D. (2010). Nitric oxide participates in the complex interplay of defense-related signaling pathways controlling disease resistance to sclerotinia sclerotiorum in arabidopsis thaliana. Mol. Plant-Microbe Interact..

[22] Williams B., Kabbage M., Kim H.-J., Britt R., Dickman M.B. (2011). Tipping the balance: Sclerotinia sclerotiorum secreted oxalic acid suppresses host defenses by manipulating the host redox environment. PLoS Pathog..

[23] Audenaert K., De Meyer G.B., Hofte M.M. (2002). Abscisic acid determines basal susceptibility of tomato to botrytis cinerea and suppresses salicylic acid-dependent signalling mechanisms. Plant Physiol..

[24] Asselbergh B., Hoefte M. (2007). Basal tomato defences to botrytis cinerea include abscisic acid-dependent callose formation. Physiol. Mol. Plant Pathol..

[25] Pieterse C.M.J., Van der Does D., Zamioudis C., Leon-Reyes A., Van Wees S.C.M. (2012). Hormonal modulation of plant immunity. Annu. Rev. Cell Dev. Biol..

[26] L’Haridon F., Besson-Bard A., Binda M., Serrano M., Abou-Mansour E., Balet F., Schoonbeek H.-J., Hess S., Mir R., Leon J. (2011). A Permeable Cuticle Is Associated with the Release of Reactive Oxygen Species and Induction of Innate Immunity. PLoS Pathog..

[27] Orozco-Cardenas M., Ryan C.A. (1999). Hydrogen peroxide is generated systemically in plant leaves by wounding and systemin via the octadecanoid pathway. Proc. Natl. Acad. Sci. USA.

[28] Govrin E.M., Levine A. (2000). The hypersensitive response facilitates plant infection by necrophytic pathogen Botrytis cinereal. Curr. Biol..

[29] Torres M.A. (2010). ROS in biotic interactions. Physiol. Plant..

[30] Guerrero-Molina M.F., Lovaisa N.C., Salazar S.M., Martinez-Zamora M.G., Diaz-Ricci J.C., Predaza R.O. (2014). Physiological, structural and molecular traits activated in strawberry plants after inoculation with the plant growth-promoting bacterium *Azospirillum brasilense* REC3. Plant Biol..

[31] Chehab E.W., Eich E., Braam J. (2009). Thigmomorphogenesis: A complex plant response to mechano-stimulation. J. Exp. Bot..

[32] Mauch F., Kmecl A., Schaffrath U., Volrath S., Gorlach J., Ward E., Ryals J., Dudler R. (1997). Mechanosensitive expression of a lipoygenase gene in wheat. Plant Physiol..

[33] Lee D., Diana H., Polisensky D.H., Braam J. (2005). Genome-wide identification of touch- and darkness-regulated Arabidopsis genes: A focus on calmodulin-like and XTH genes. New Phytol..

[34] Tretner C., Huth U., Hause B. (2008). Mechanostimulation of *Medicago truncatula* leads to enhanced levels of jasmonic acid. J. Exp. Bot..

[35] Mitchell C.A., Myers P.N. (1995). Mechanical stress regulation of plant growth and development. Hortic. Rev..

[36] Coutand C. (2010). Mechanosensing and thigmomorphogenesis, a physiological and biomechanical point of view. Plant Sci..

[37] Jehong Y., Ota Y. (1990). A relationship between growth inhibition and abscisic acid content by mechanical stimulation in rice plant. Jpn. J. Crop Sci..

[38] Erner Y., Jaffe M.J. (1982). Thigmomorphogenesis: The involvement of auxin and abscisic acid in growth retardation due to mechanical perturbation. Plant Cell Physiol..

[39] Cipollini D.F., Redman A.M. (1999). Age-dependent effects of jasmonic acid treatment and wind exposure on foliar oxidase activity and insect resistance in tomato. J. Chem. Ecol..

[40] Cipollini D.F. (1998). The induction of soluble peroxidase activity in bean leaves by wind-induced mechanical perturbation. Am. J. Bot..

[41] Thomma B.P.H.J., Eggermont K., Penninckx I.A.M.A., Mauch-Mani B., Voselsang R., Cammue B.P.A., Broekaert W.F. (1998). Separate jasmonate-depedent and salicylate-dependent defense-response pathways in Arabidopsis are essential for resistance to distinct microbial pathogens. Proc. Natl. Acad. Sci. USA.

[42] Botella J.R., Arteca R.N., Frangos J.A. (1995). A mechanical strain-induced 1-aminocyclopropane-1-carboxylic acid synthase gene. Proc. Natl. Acad. Sci. USA.

[43] Kissoudis C., Sunarti S., van de Wiel C., Visser R.G.F., van der Linden G., Bai Y. (2016). Responses to combined abiotic and biotic stress in tomato are governed by stress intensity and resistance mechanism. J. Exp. Bot..

[44] Kissoudis C., Seifi A., Yan Z., Islam A.T.M.T., van der Schoot H., van de Wiel C.C.M., Visser R.G.F., van der Linden C.G., Bai Y. (2017). Ethylene and Abscisic Acid Signaling Pathways Differentially Influence Tomato Resistance to Combined Powdery Mildew and Salt Stress. Front. Plant Sci..

[45] Suzuki N., Rivero R.M., Shulaev V., Blumwald E., Mittler R. (2014). Abiotic and biotic stress combinations. New Phytol..

[46] Bai Y., Sunarti S., Kissoudis C., Visser R.G.F., van der Linden C.G. (2018). The Role of Tomato *WRKY* Genes in Plant Responses to Combined Abiotic and Biotic Stresses. Front. Plant Sci..

[47] Van Loon L.C., Rep M., Pieterse C.M. (2006). Significance of inducible defense-related proteins in infected plants. Annu. Rev. Phytopathol..

[48] Graham M.Y., Weifer J., Wheeler K., Pelow M.J., Graham T.L. (2003). Induced expression of pathogenesis-related protein genes in soybean by wounding and the Phythophthora sojae cell well glucan elicitor. Physiol. Mol. Plant Pathol..

[49] Conrath U. (2006). Systemic acquired resistance. Plant Signal. Behav..

[50] Ishihara K.L., Eric K.W., Lee E.K.W., Borthakur D. (2017). Thigmomorphogenesis: Changes in morphology; biochemistry; and levels of transcription in response to mechanical stress in Acacia koa. Can. J. For. Res..

[51] Gus-Mayer S., Naton B., Hahlbrock K., Schmelzer E. (1998). Local mechanical stimulation induces components of the pathogen defense response in parsley. Proc. Natl. Acad. Sci. USA.

[52] Wang S.L., Liu C.P., Liang T.W. (2012). Fermented and enzymatic production of chitin/chitosan oligosaccharides by extracellular chitinases from *Bacillus cereus* TKU027. Carbohydr. Polym..

[53] Diaz J., ten Have A., van Kan J.A.L. (2002). The role of ethylene and wound signaling in resistance of tomato to botrytis cinerea. Plant Physiol..

[54] Dardouri T., Gautier H., Ben Issa R., Costagliola G., Gomez L. (2018). Repellence of Myzus persicae (Sulzer): Evidence of two modes of action of volatiles from selected living aromatic plants. Pest Manag. Sci..

[55] Dardouri T., Gomez L., Dcheny A., Costagliola G., Gautier H. (2019). Behavioural response of green peach aphid Myzus persicae (Sulzer). to volatiles from different rosemary (Rosmarinus officinalis L.) clones. Agric. For. Entomol..

[56] Bengtsson G.B. (2010). Effect of Postharvest Conditions and Treatments on Health-Related Quality of Vegetables and Fruits. III INTERNATIONAL CONFERENCE POSTHARVEST UNLIMITED 2008. Acta Hortic..

